# Repair of osteochondral defect using icariin-conditioned serum combined with chitosan in rabbit knees

**DOI:** 10.1186/s12906-020-02996-3

**Published:** 2020-06-22

**Authors:** Juntao Zhang, Dong Ming, Qiang Ji, Aifeng Liu, Chao Zhang, Jianjie Jiao, Man Shang

**Affiliations:** 1 Academy of Medical Engineering and Translational Medicine, Tianjin University, 92 Weijin Road, Nankai district, Tianjin, China; 2 Department of orthopedics, First Teaching Hospital of Tianjin University of Traditional Chinese Medicine, 88 Changling Road, Xiqing district, Tianjin, China; 3 Tianjin University of Traditional Chinese Medicine, 10 Boyanghu Road, Jinghai district, Tianjin, China; 4 Department of Pharmacology, School of Basic Medical Sciences, Tianjin Medical University, 22 Qixiangtai Road, Heping District, Tianjin, China

**Keywords:** Cartilage defect, Chondrocyte, Icarrin, Chitosan

## Abstract

**Background:**

Osteochondral defects caused by an acute traumatic injury or articular degeneration remains difficult to be manipulated. Repair of articular defects is still a great challenge for both tissue engineers and orthopedic surgeons. Therefore, combination of biomaterials with cartilage promotive drugs is well worth being developed to support the regeneration of both cartilage and subchondral bone.

**Methods:**

Rabbits undergoing osteochondral defect surgery were intrarticularly injected with icariin-conditioned serum (ICS), chitosan (CSSH) and combination of ICS with CSSH, respectively. Gait analysis was performed using VICON motion capture system. ICRS score and immunohistochemical (IHC) analysis including H&E, Safranin O, toluidine blue and collagen II staining was employed to evaluate macroscopic cartilage regeneration and determine the morphologic repair of cartilage.

**Results:**

Rabbits with the treatment of ICS or CSSH alone showed mild improvement in hopping time and range of joint angles while ICS-CSSH group exhibited longer jumping time and larger range of joint angles. In addition, femoral condyle in ICS-CSSH rabbits could be seen with more native cartilage and subchondral bone regeneration in both macroscopic observation and IHC analysis.

**Conclusion:**

ICS combined with CSSH could promote the repair of osteochondral defect in rabbit knees. Combination of biomaterials with cartilage promotive drugs may ultimately have profound implications in the management of cartilage defect.

## Background

An osteochondral defect is lesions of disruption to articular cartilage and underlying (subchondral) bone. Usually, osteochondral defects appear on specific weight-bearing spots at the ends of the thighbone and shinbone and the back of the kneecap. These can occur from an acute traumatic injury to the joint or an underlying disorder of the bone. Articular cartilage has no blood supply and its ability to repair itself is poor. Studies have shown that these lesions tend to progress in size and severity, affecting the rest of the articular cartilage and predisposing you to suffer from an early onset of arthritis. The present treatments for osteochondral defect are limited and insufficient to prevent the progression of the disease. Therefore, it is needed to explore new strategies for the prevention and treatment of osteochondral defect.

Current therapies include NSAIDs (nonsteroidal anti-inflammatory drugs), physical therapy and exercise, excision surgery to remove the lesion area. Treatment options to restore joint congruity vary widely from nonoperative closed treatment to arthroscopic drilling, with or without fixation, to tissue transplantation or reconstructive procedures. However, there is no effective treatment for cartilage defect but only strategies for symptom relief. Stimulating the formation of cartilage to replace the original cartilage defect has aroused our attention to explore new approaches to challenging osteochondral defect. Modalities like tissue engineering with combined materials and drug-based therapy are more and more widely used.

Recently, an innovative therapy by autologous conditioned serum (ACS) from the whole blood was settled [1]. Meijer et al. first devised this biologic therapeutic preparation ACS, marketed as ‘Orthokinew’ (Orthogen, Düsseldorf, Germany), to produce ACS enriched with anti-inflammatory cytokines [2]. Frizziero’ research has showed some potentially responders considered to apply ACS intra-articular injections for patients with a risk of osteoarthritis development [3]. This biological treatment has been assayed in veterinary medicine for the treatment of osteoarthritis in horses [4]. Clinical trials have demonstrated its beneficial effect in knee osteoarthritis [5, 6]. Thus, conditioned serum has been used intra-articularly for osteoarthritic patients possibly attributing to its rich content on anti-inflammatory proteins and growth factors [7]. ACS is proposed to be capable of leading to improvement of tissue regeneration and to reduction of degenerative mechanisms.

Based on novel opinions, we have prepared rabbit conditioned serum with icariin in the previous research [8]. Icariin is a prenylated flavonol glycoside isolated from a very used traditonal Chinese medicine *Epimedium* herb, which has been shown to be the main bioactive component and more effective than other flavonoid compounds in facilitating chondrocyte vitality and promoting articular cartilage repair [9, 10]. Thus, we prepared icariin-conditioned serum (ICS) in rabbits and have demonstrated the proliferation effects of ICS on primary rabbit chondrocytes in vitro and osterochondral defect models in vivo [8].

Chitosan, a deacetylated derivative from chitin is an important natural polymer that is widely used for cartilage repair in combination with other natural or synthetic polymers [11–14]. It has been reported that chitosan has various important pharmacological properties especially with well documented roles in tissue engineering and regenerative medicine. Chitosan based hydrogels have been used greatly in the field of medicine including delivery of nucleic acid, wound healing, neural and vascular tissue engineering, and dental implants [15–17]. The inherent antimicrobial properties, biodegradability and biocompatibility makes chitosan a popular choice for tissue engineering applications. It has been reported that chitosan-based composite bilayer scaffold is beneficial for the proliferation and migration of chondrocyte-like cells SW-1353 to promote osteochondral tissue regeneration in vitro [18]. Chitosan-hyaluronic acid dialdehyde hydrogels enabled osteochondral defects in rabbit models showed texture similar to the surrounding native cartilage [19]. It is considered that using chitosan as the support for tissue engineering is promising in cartilage regeneration. Therefore, it is proposed that the inoculation of ICS into chitosan solutionwill reveal promotive efficacy on the repair of osteochondral defect.

To test the hypothesis, critical size osteochondral defects were created in knee joints of Newzealand White rabbits to investigate the suitability of ICS combined with chitosan in vivo. Our study showed chitosan with low toxicity in cultured cells and ICS combined with chitosan was highly effective for the enhancement of cartilage regeneration and osteochondral defect repair.

## Method

### Animals

New Zealand White Rabbits were purchased from Bejing Charles River Laboratories. Young rabbits (4 weeks) were assigned for collection of primary chondrocytes while adult rabbits (12 weeks) for preparation of icariin conditioned serum (ICS) and models of osteochondral defect in knees. All animals were kept in specific pathogen free (SPF) enviroment and have free access to food and water. Animal experiment protocols were performed in accordance with the Declaration of Helsinki of the World Medical Association and the research was approved by Ethics Committee of Tianjin University of TCM (TCM-LAEC20170026).

### Preparation of thiolated chitosan (CSSH)

Take 500 mg of chitosan (Sangon, Shanghai, China) and disperse it in 46 mL of distilled water, and stir for 5 min to make the chitosan evenly dispersed. Then add 348.6 mg of HOBT (Sangon, Shanghai, China) and stir for 30 min. After the solution becomes clear, add 842 mg of NAC to it and stir for 5 min to disperse the NAC (Sangon, Shanghai, China) evenly. Then add EDCI-HCl (Sigma, St. Louis, MO, USA) solution (1978.5 mg of EDCI-HCl dissolved in 4 mL of distilled water). After the solution becomes clear, stir for 5 min and measure the solution pH. If the pH is greater than 5, add a 1 M hydrochloric acid solution to it, adjust the pH to about 5, and stir for 7 h. The reaction product was put into a dialysis bag with Mw = 7000, and dialyzed with a distilled aqueous solution containing 5 mM hydrochloric acid and 2 μM EDTA for 3 days, and then dialyzed with a distilled aqueous solution containing 5 mM hydrochloric acid, 2 μM EDTA and 0.1% NaCl for 2 days. Next the solution containing 5 mM hydrochloric acid was used to dialyze for 1 day, and finally the distilled water was used to dialyze for 1 day. All dialysis processes are performed at 4 °C in dark. The dialysis products are lyophilized and sealed.

### Cell culture and cytotoxicity assay of CSSH

Mouse fibroblast L929 was obtained from ATCC and cultured in dulbecco’s modified eagle medium (DMEM, Gibco, USA) supplemented with 10% FBS in a humidified atmosphere of 5% CO_2_ at 37 °C. Thiolated chitosan was synthesized by Tianjin University [20] and detected using nuclear magnetic resonance (NMR). To make sure CSSH could be used as injectablesolution, its solubility should be at 0.75 mg/ml. CSSH was dissolved in DMEM, diluted in the concentration of 0.75, 1.5, 3 mg/ml, and filtered with 0.2 μm strainer. For cytotoxicity assay of CSSH, cells treated by 10% DMSO were used as positive control. Methyl thiazolyl tetrazolium (MTT, Amresco, Solon, OH, USA) assay was used to detect the cytotoxicity of CSSH. L929 cells were plated in 96-well plates at a density of 6 × 10^4^ cells/mL and cultured with CSSH in indicated concentrations in DMEM for 24–72 h, respectively. At the end of treatment, 10 μL 0.5% MTT solution was added to cells and incuated at 37 °C for 4 h. The cytotoxicity was determined by the absorbance of the blue formazan derivative dissolved by 150 μL DMSO. The OD value was measured at 490 nm by a microplate reader (Bio-Rad Laboratories, CA, USA).

### Collection of ICS

Icariin was purchased from Shanghai Ronghe pharmaceutical technologyco. LTD (Lot:160602, purifity≥98%). Adult rabbits were administered with icariinto prepare ICS for further administration on animal models of cartilage defects. The equivalent dose of icariin on rabbits calculated according to human is 0.47 g/kg. Twenty rabbits were orally administered with icariin at the dose of 0.94 g/kg everyday for 1 week [8]. Rabbits were anesthetized using urethane 2 h after the last administration. Circulating blood was collected from abdominal artery and centrifuged at 2000 g, 10 min. ICS was filtered with 0.22 μm strainer and freezed to dried powder by FD5–2.5 Freeze Dryer (SIM, Newark, USA) which could be freely soluble in normal saline or CSSH injection.

### Animal model and treatment

Forty-eight male New Zealand White rabbits weighing between 2.8 and 3.3 kg were used. Rabbits were operated on both knees to be established with osteochondral defect models. Precise surgery procedure has been described previously [8]. The size and depth of the articular cartilage defects was made at a diameter of 4 mm and a depth of 3 mm [21]. All animals were kept in individual cages and allowed to move freely after surgery. There were no complications encountered during the performance of the operation or in the postoperative recovery period. Rabbits randomly assigned in different goups were intra-articularly injected with 0.5 mL NS, ICS, CSSH and ICS-CSSH in the right knee joint, respectively. Each groups consisted of 12 rabbits. Intra-articular injection was started at the beginning of third week after surgery and lasted for five weeks. Twenty-four rabbits were euthanized with 120 mg/kg of sodium pentobarbital (MTC Pharmaceuticals, Ontario, Canada) 8 and 12 weeks after surgery, respectively. Knee joints were retrieved for macroscopic evaluation and histological analysis.

### Motion capture analysis

Motion capture analysis on time of jumping cycle and joint angles were performed on rabbits before surgery, 8 and 12 weeks after surgery. The motion data of rabbits were collected at 200 Hz with a 15-camera motion capture system (Vicon Motion Systems Ltd., Oxford, UK). Nine reflective makers were stuck on the phalanges, joint of tarsal bone and the tibia, joint of the tibia and femur and termination of femur, and one was stuck on the middle of spine. Joint angles and jumping time were calculated from the motion data. Jumping trials of the rabbits were selected for the analysis. The time of jumping cycle was extracted according to the motion data. The joint angles of left ankle and right ankle when rabbit jumping from slope top to the bottom were calculated.

For the calculation of jumping time, the reflective markers at the rabbit’s foot was chosen. Time of the rabbit foot jumping from the ground to the ground again was determined as the jumping time according to the change of the Z-axis. Relective markers L1, L2, L3, L4 on the left side of the rabbit were chosen as an example to calculated joint angles. The angle a1 between the vector L2L1 and the vector L2L3 is considered as the angle of ankle joint, and the angle a2 between the vector L3L2 and L3L4 is considered as the angle of knee joint. For the calculation of ankle joint angle, the degree a1 of ankle joint was firstly determined, and then the difference between the maximum and minimum values of the ankle joint angle was indicated as the change of the joint angle range.

### Macroscopic evaluation of repair on osteochondral defect in vivo

After 8 and 12 weeks observation, the retrieved knee joints were photographed and the synovium was observed for signs of inflammation, and the joint itself was inspected for osteophyte formation indicating degeneration. The macroscopic evaluation consists of the surface characteristics of the regenerated tissue and their integration with the surrounding tissue were blindly examined using the International Cartilage Repair Society (ICRS) macroscopic assessment scale as shown in supplemental Table 1 [22, 23].

### Histological and immunohistochemical analysis

The retrieved joint samples were conducted following routine paraffin sections. Slides were stained with Hematoxylin and eosin (H&E), toluidine blue and safranin O for morphological observation. They were also immunized with anti-collagenII primary antibody (1/400 diluted, Bioss, Beijing, China) and stained with diaminobenzidine peroxidase substrate. The slides were observed under a upright microscope (Nikon, Japan). Makin scores were evaluatedaccording to four parameters including cartilage structure, cellularity, Safranin O staining and tidemark integrity [24].

### Statistical analysis

Data were presented as mean ± SD by using Prism 7.0 software (GraphPad Prism). Differences between two groups were analyzed by independent-sample student’s t-test. Results were considered statistically significant when *P* < 0.05.

## Results

### CSSH showed no cytotoxic effects on L929

**Fig. 1:**
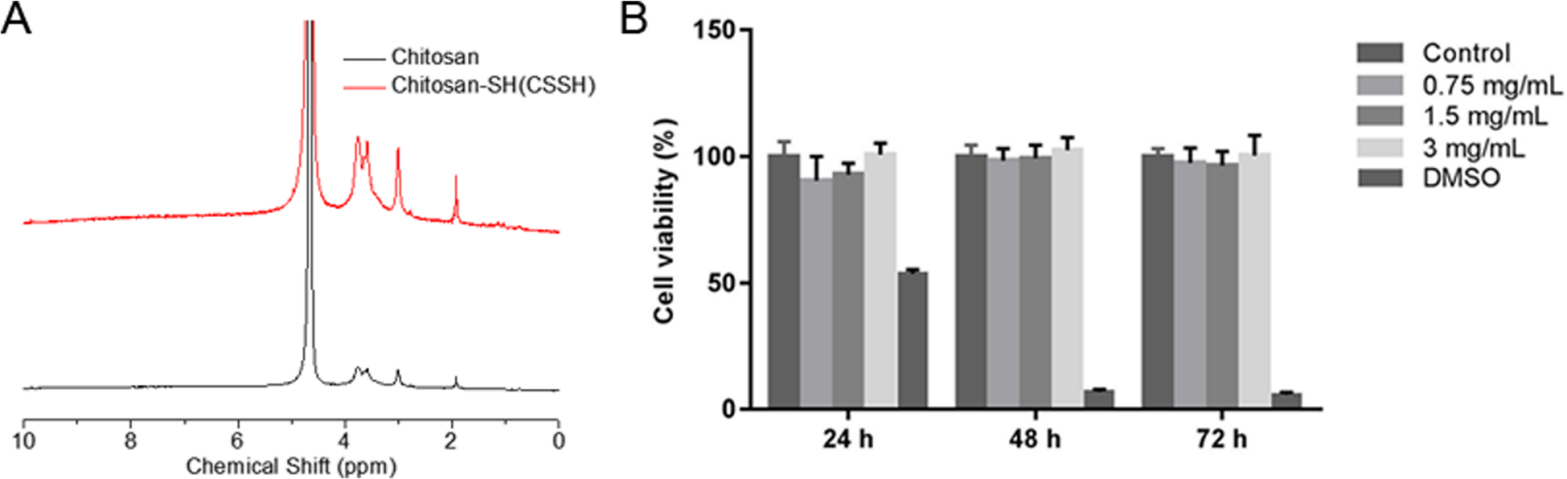
CSSH showed no cytotoxic effects on L929 cells. **a** NMR spectrogram for chitosan and chitosan-SH (CSSH). **b** Cell viability of L929 fibroblasts treated by CSSH at the concentrations of 0.75, 1.5, 3 mg/ml for 24, 48, 72 h (*n* = 8, mean ± SD)

CSSH was synethized, and the obtained CSSH can be well soluted in water. It exhibited the similar main peak with chitosan, as shown by the NMR spectrogram. (Fig. 1a). The cytotoxicity of CSSH was evaluated by MTT assay at 24, 48 and 72 h on L929 fibroblasts. The results showed cell viability of L929 could maintain at more than 90% (Fig. 1b). No significant changes in CSSH treatment was observed at the concentrations of 0.75, 1.5, 3 mg/ml. Thus, CSSH should be safe without cytotoxicity at least at the concentration of 3 mg/ml.

### ICS-CSSH improved gait performance on rabbits with osteochondral defects

**Fig. 2:**
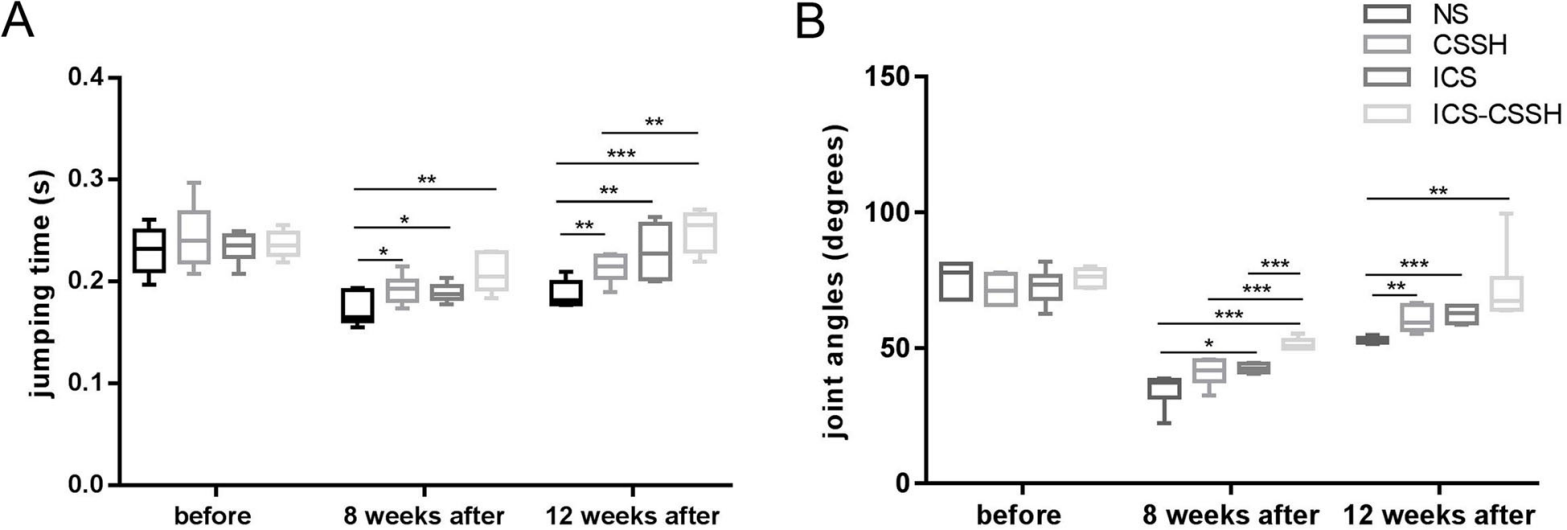
ICS-CSSH improved gait performance on rabbits with osteochondral defects. **a** and **b** Time of jumping cycle and joint angles were detected using motion capture system on rabbits before surgery, 8 and 12 weeks postoperation (*n* = 6, mean ± SD). *P < 0.05, **P < 0.01, ***P < 0.001 vesus indacated groups

Time of jumping cycle and joint angles were detected using motion capture system on rabbits before surgery, 8 and 12 weeks postoperation. Both jumping time and joint angles were almost the same before the establishment of osteocondral defect models. At the end of 8 weeks after surgery, ICS-CSSH rabbits showed prolonged time of jumping cycle and increased joint angles, indicating better recovery from the damage of cartilage defect (*P* < 0.01, Fig. 2a, b). ICS and CSSH group also showed improvement on gait but not as good as ICS-CSSH. At the end of 12 weeks postoperation, gait in all treatment groups except NS were further improved, showing rabbits could move more freely without claudication (Fig. 2a, b).

### ICS-CSSH promoted the regeneration of cartilage defect in vivo through macroscopic observation

**Fig. 3:**
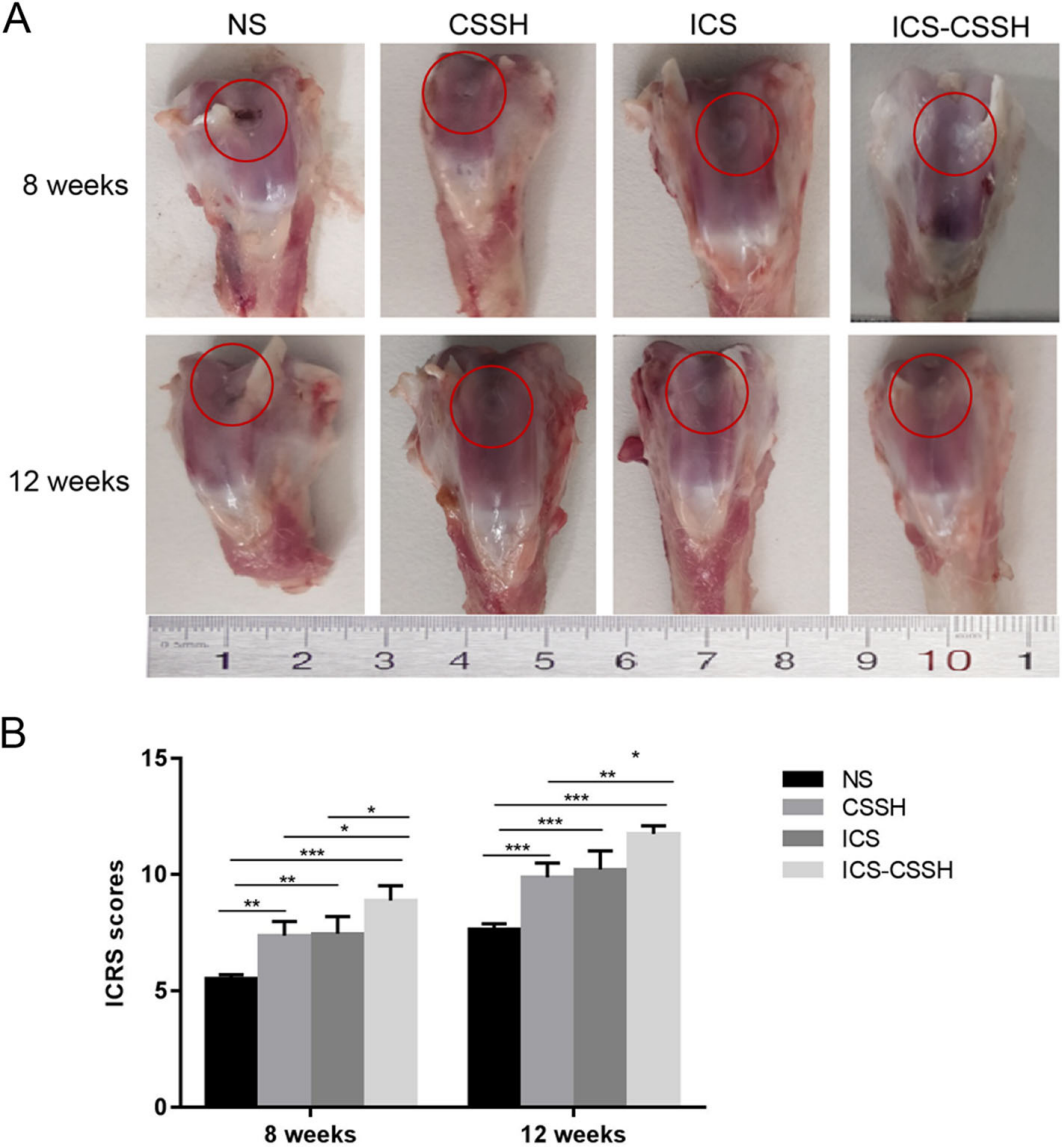
ICS-CSSH promoted the regeneration of cartilage defect in vivo through macroscopic observation. **a** Macroscopic observation of cartilage defect in NS, CSSH, ICS and ICS-CSSH groups. The cartilage defect area was labeled in the red circle. The cartilage surface of the femoral condyle in defect area could be compared with the surrounding area to evaluate its general state of repair. **b** ICRS scale for cartilage regeneration in NS, CSSH, ICS and ICS-CSSH groups (n = 6, mean ± SD). ICRS, international cartilage repair society. *P < 0.05, **P < 0.01, ***P < 0.001 vesus indacated groups

After surgery for 8 weeks, the changes of cartilage defects in rabbits in each group were observed by gross morphology in terms of the surface of the defect,surrounding cartilage and exposure of the subchondral bone. The recovery of the defect area of the right knee joint of rabbits in each group was different. In the NS group, the surface of the defect area was uneven, the shape was not completely repaired, and the cartilage surface of the femoral condyle was incomplete. The exposed subchondral bone was visible. The cartilage surface of the knee joint in the CSSH group and the ICS group was nearly complete, but the surface of the articular cartilage was slightly uneven. In the ICS-CSSH group, the cartilage surface defect area of the knee joint was smooth and milky white. The repair of the shape was nearly complete, and the degree of cartilage degeneration in the joint was lighter (Fig. 3a).

After surgery for 12 weeks, the cartilage defect area in each group was observed to further recover. The defect site in the NS group was still sunken, but nearly flat. In the CSSH group and the ICS group, the repair of the defect surface was basically flat. The defect area of the cartilage surface of the knee joint in the ICS + CSSH group was smooth and better than CSSH or ICS group (Fig. 3a).

The defect area was analyzed histologically using the ICRS scale for cartilage regeneration. The ICRS score showed a significantly higher score in the treatment groups compared with the NS group (*P* < 0.05, Fig. 3b). Among them, the cartilage defect repair effect was better in the ICS-CSSH group than in the other treatment groups (P < 0.05, Fig. 3b). The higher the ICRS score in the ICS-CSSH group showed better recovery of cartilage defects.

### ICS-CSSH promoted the regeneration of cartilage defect regeneration in vivo through histologic observation

**Fig. 4:**
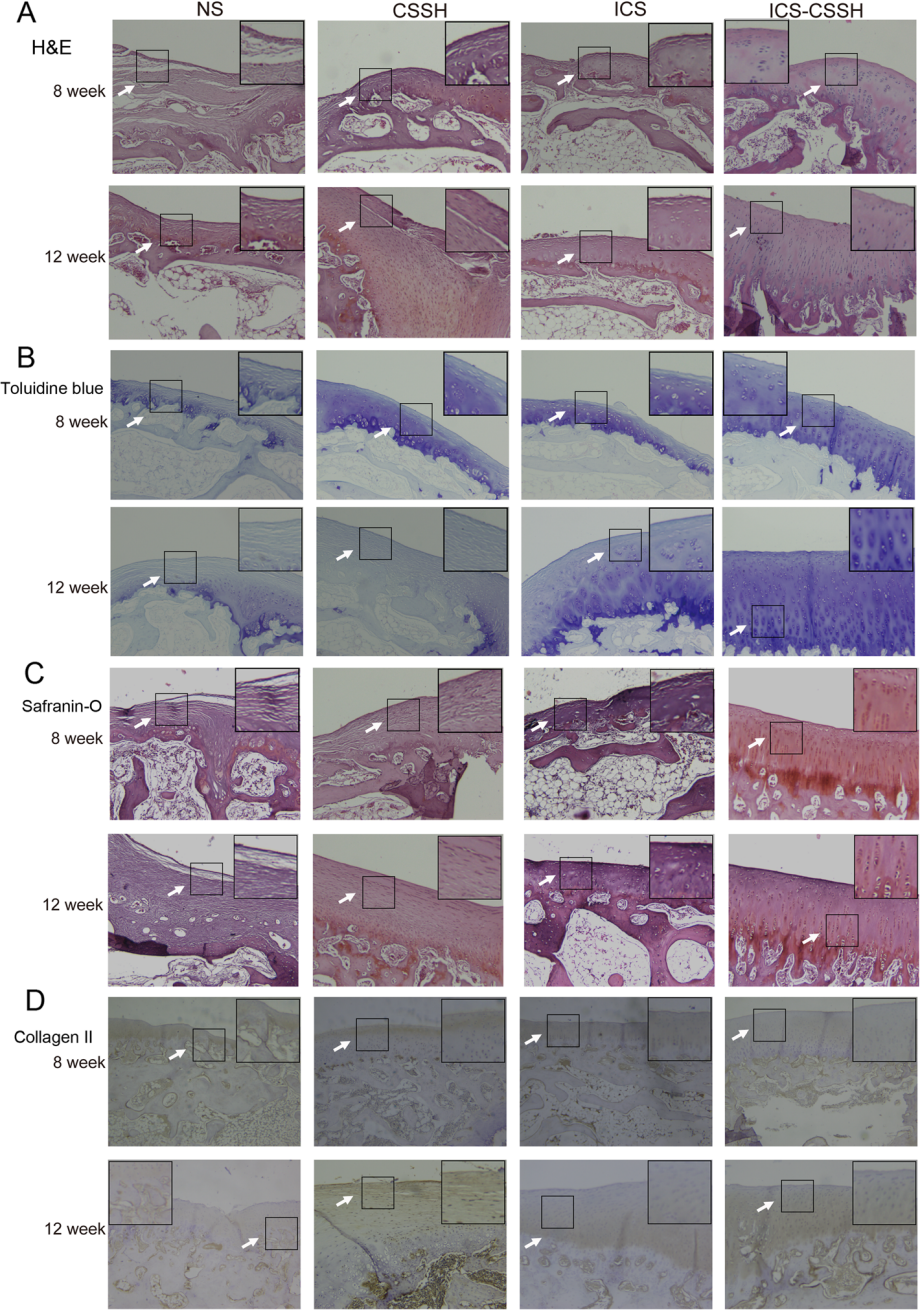
ICS-CSSH promoted the regeneration of cartilage defect regeneration in vivo through histologic observation. **a**-**d** Histological and immunohistochemical analysis of osteochondral defects repair in rabbit knees

Throughmicroscopy it can be clearly seen that articular cartilage is divided into tangent layer or surface layer, transfer layer, radiation layer and cartilage matrix calcification layer from shallow to deep. 8 weeks after surgery, the cartilage defect area of the femoral condylestained by H&E in NS showed extensive loss of cartilage or extensive fibrosis, the defect was filled with fibrous granulation tissue and irregularly arranged chondrocytes. There were very few cells in the transition layer and radiation layer, and the low tide line disappears. The repair of articular cartilage defects in the CSSH group showed cracked cells, irregular arrangement and unclear tide line. The morphology of the repaired cells in the ICS group was slightly different from normal cartilage, and thetide line was difficult to observe. In the ICS + CSSH group, the cartilage defect area of the femoral condyle was gradually repaired by cartilage-like tissue. The surface of the repaired area was smooth and cracks were rare. The chondrocytes in the repaired area gradually approached the shape of normal articular chondrocytes, the arrangement tended to be regular, and the structure was clearer. The tide line gradually recovered (Fig. 4a).

Twelve weeks after the operation, the defect in the NS group had been filled with fibrous granulation tissue, and the low tide line had not recovered. In the CSSH group, the fissures in the articular cartilage defect still existed and thetide line was difficult to observe. The tidal line of the ICS group was gradually restored. In the ICS + CSSH group, the defect area recovered well, basically close to normal chondrocytes, the surface of the repaired area was smooth, and the low tide line recovered (Fig. 4a). In addition, each group of specimens was scored by HE histology using Mankin method (supplemental Figure S2). ICS-CSSH with the lowest scores showed better recovery from the cartilage defect. Toluidine blue staining for chondrocytes showed more hyaline cartilage in ICS-CSSH group than other groups. Moreover, Safranin-O staining for GAG, immunehistochemical staining for collagen II also demonstrated that the newly formed tissue was predominantly hyaline cartilage (Fig. 4b-d).

## Discussion

Because of the avascular property and confinement of resident chondrocytes to dense extracellular matrix, adult cartilage has a very limited healing capacity after injury. Healing of osteochondral defects remains a clinical challengewith significant research endeavor directed at optimizing treatment strategies. The present study has showed the promotive effects of ICS-CSSH on the formation of hyaline cartilage and repair of subchondral bone. On the basis of present findings reported here, drug-loaded compatible materials could be considered as a new strategy for the improvement of healing capacity of cartilage defects.

In the present study, chitosan was emplyed to serve as a biomaterial supporting the repair process. Our results showed thiolated chitosan was alomost non-toxic to cells, it should be safe as a biomaterial for tissue engineering. CSSH used in the animal experiments also showed certain effect on the repair of cartilage repair. Evidence suggests that chitosan can be a favorable drug delivery system because of its unique properties [25]. Previous researches have stated chitosan is an important natural ploymer that is widely used for cartilage repair with other natural or synthetic polymers. Chitosan composed of glucosamine and *N*-acetylglucosamine, is structurally similar to glycosaminoglycans (GAG), present in extra cellular matrix of cartilage. Thus, chitosan and its derivatives such as CSSH could be widely used for drug delivery, and tissue engineering applications because of the favorable structural features and biodegradability.

Drug conditioned serum has been used in our previous study and confirmed to be partially effective in the recovery [8]. Icariin, as a flavonoid constituent isolated from Epimedium Herb, has been researched extensively in the field of regenerative medicine, it has been demonstrated to be capable of enhancing osteogenic differentiation, facilitating matrix calcification and inhibiting osteoclastic bone resorption with or without inductive medium [26–31]. Meanwhile, other studies pointed out that icariin could promote proliferation and maintain the phenotype of chondrocytes, increase the secretion of proteoglycan and collagen matrix by chondrocytes, and inhibit the degradation of collagen and proteoglycan. ICS is extracted from rabbit gavaged with icariin. Pharmacokinetic studies showed icariin could be metabolized by intestinal flora and converted to its derivatives like icaritin, icariside I, icariside II, and desmethylicaritin [32, 33]. In this way, conditioned serum containing icariin metabolites could exert cartilage promotive effects. In addition, some prospective randomized controlled trials have considered ACS as an interesting, well-tolerated and possibly effective option in human knee osteoarthritis (OA) [34]. Plate rich plasma (PRP) was also be used to improve cartilage repair as an injectable implants [35, 36]. Although the exact mechanism of PRP action has not been understood, PRP could positively influence expression of cartilage matrix, and migration, proliferation, chondrogenesis of subchondral bone marrow stem cells (BMSCs), as it is s a rich source of growth factors and cytokines, which play important roles in inflammatory and wound repair phenomena [37]. Taken together, it should be prospective to attempt the combination of drug conditioned serum with chitosan. Our results indeed showed Safranin O staining of defect area in ICS-CSSH group is increased together with stronger toluidine blue and collagen II staining of newly formed cartilage and subchondral bone formation.

Numerous experimental investigations on the repair of osteochondral defects with tissue-engineered cartilage constructs have been conducted which show superior effects compared to the natural healing capacity of cartilage injuries. However, there is no consensus regarding the most effective form of treatment. Understanding the mechanisms behind normal healing is vital to inform therapy selection and experimental design. Our finding provides information that may assist the design of drug loading and tissue engineering strategies to heal osteochondral defects. However, very limitated information of ICS is available for further exploration. This finding also casts the need for exploring precise mechanism on the acutual composition of drug conditioned serum and further applications of high biocompatible engineering materials.

## Conclusions

ICS and CSSH could partially promote osteochondral defect repair with incomplete integration of cartilage superficial layer and subchondral bone, but ICS-CSSH showed strong capacity in the enhancement of cartilage regeneration and subchondral bone formation. The combination application of drug conditioned serum and chitosan can be considered as new strategies for repair of osteochondral defect and even tissue engineering.

## Supplementary information

**Additional file 1: Table S1**. ICRS macroscopic evaluation of cartilage repair.

**Additional file 2: Figure S1**. Diagram for the calculation of joint angles.

**Additional file 3: Figure S2**. Markin scores for the evaluation of H&E staining in NS, CSSH, ICS and ICS-CSSH groups. *P < 0.05, **P < 0.01 vesus indacated groups.

## Data Availability

The datasets analyzed during the current study are available from the corresponding author on reasonable request. All data supporting the conclusions are included in the article and Additional files (supplemental Tables S1, Figure S1, Figure S2).

## Abbreviations

ACS: Autologous conditioned serum
BMSCs: Bone marrow stem cells
CSSH: Thiolated chitosan
DMEM: Dulbecco’s modified eagle medium
GAG: Glycosaminoglycans
H&E: Hematoxylin & eosin
ICA: Icariin
ICRS: International cartilage repair society
ICS: Icariin conditioned serum
MTT: Methyl thiazolyl tetrazolium
NMR: Nuclear magnetic resonance
NS: Normal saline
NSAIDs: Nonsteroidal anti-inflammatory drugs
OA: Osteoarthritis
PBS: Phosphate buffer saline
PRP: Plate rich plasma
SPF: Specific pathogen free
