# Correction: Repair of osteochondral defect using icariin-conditioned serum combined with chitosan in rabbit knees

**DOI:** 10.1186/s12906-022-03821-9

**Published:** 2022-12-22

**Authors:** Juntao Zhang, Dong Ming, Qiang Ji, Aifeng Liu, Chao Zhang, Jianjie Jiao, Man Shang

**Affiliations:** 1 Academy of Medical Engineering and Translational Medicine, Tianjin University, 92 Weijin Road, Nankai district, Tianjin, China; 2 Department of Orthopedics, First Teaching Hospital of Tianjin University of Traditional Chinese Medicine, 88 Changling Road, Xiqing district, Tianjin, China; 3 Tianjin University of Traditional Chinese Medicine, 10 Boyanghu Road, Jinghai district, Tianjin, China; 4 Department of Pharmacology, School of Basic Medical Sciences, Tianjin Medical University, 22 Qixiangtai Road, Heping District, Tianjin, China

**Correction: BMC Complement Med Ther 20, 193 (2020)**

**https://doi.org/10.1186/s12906-020-02996-3**

Following publication of the original article [1], the authors reported an error in Fig. 4D. The immunohistological image of collagen II (12 weeks, ICS-CSSH) was in different magnification from other images. The correct figure is given below.

**Fig. 4:**
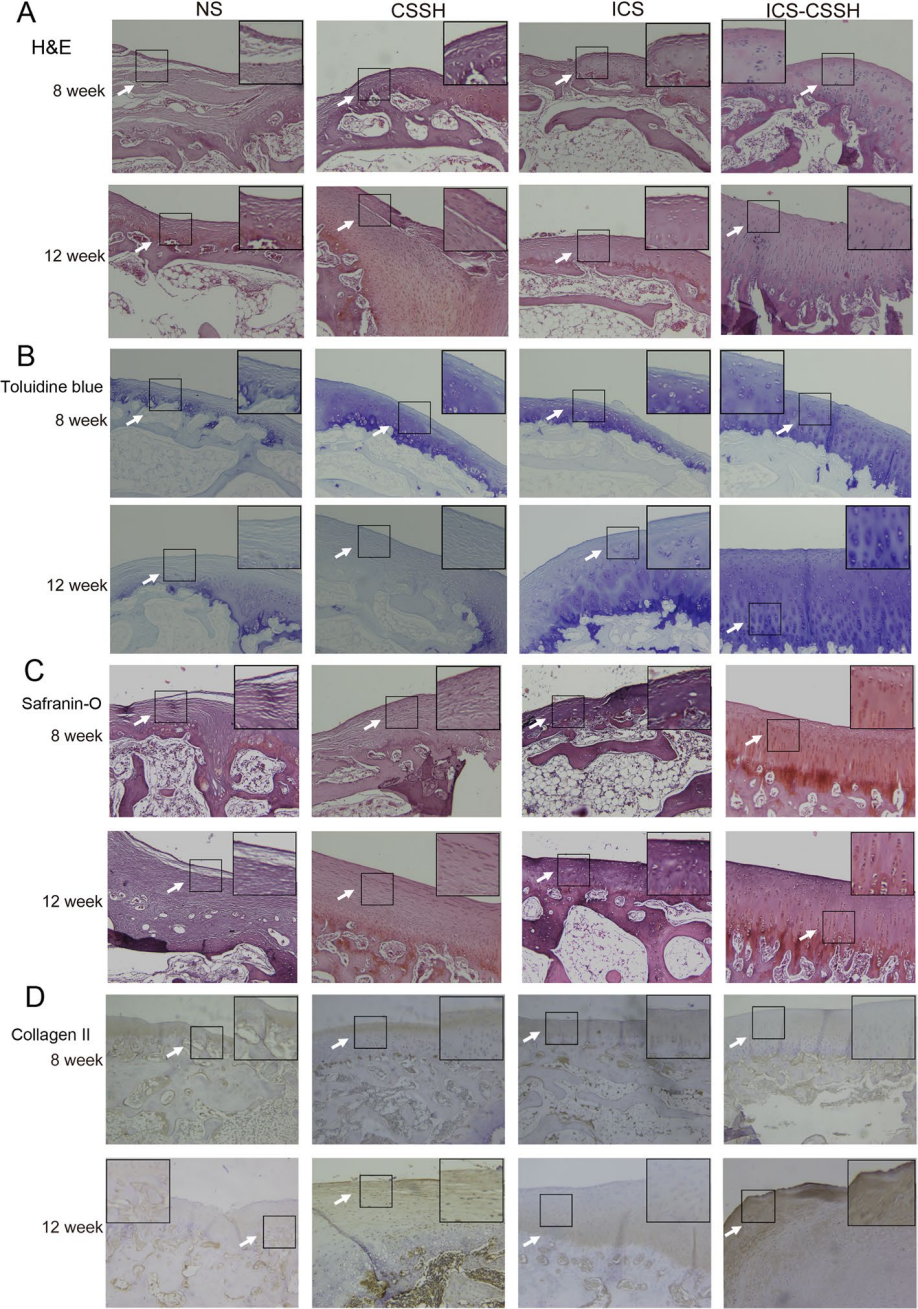
ICS-CSSH promoted the regeneration of cartilage defect regeneration in vivo through histologic observation. **a**-**d** Histological and immunohistochemical analysis of osteochondral defects repair in rabbit knees

The original article [1] has been updated.
