# Supplementary material for: Repair of osteochondral defect using icariin-conditioned serum combined with chitosan in rabbit knees
Source: BMC Complement Med Ther. 2020 Jun 22;20:193. doi: 10.1186/s12906-020-02996-3 (PMC7310103; doi:10.1186/s12906-020-02996-3)
Supplement: Supplementary file 1 — Additional file 1: Table S1. ICRS macroscopic evaluation of cartilage repair. [file 12906_2020_2996_MOESM1_ESM.pdf]

Table S1

ICRS macroscopic evaluation of cartilage repair

Cartilage repair assessment ICRS	Points
Degree of defect repair	
In level with surrounding cartilage	4
75% repair of defect depth	3
50% repair of defect depth	2
25% repair of defect depth	1
0% repair of defect depth	0
Integration to border zone	
Complete integration with surrounding cartilage	4
Demarcating border <1 mm	3
3/4th of graft integrated, 1/4th with a notable border > 1 mm width	2
1/2 of graft integrated with surrounding cartilage, 1/2 with a notable border >1 mm	1
From no contact to 1/4th of graft integrated with surrounding cartilage	0
Macroscopic appearance	
Intact smooth surface	4
Fibrillated surface	3
Small, scattered fissures or cracs	2
Several, small or few but large fissures	1
Total degeneration of grafted area	0
Overall repair assessment	
Grade I: normal	12
Grade II: nearly normal	11-8
Grade III: abnormal	7-4
Grade IV: severely abnormal	3-1

Abbreviations: ICRS, international cartilage repair society.
