# Supplementary figures and images for: Repair of osteochondral defect using icariin-conditioned serum combined with chitosan in rabbit knees
Source: BMC Complement Med Ther. 2020 Jun 22;20:193. doi: 10.1186/s12906-020-02996-3 (PMC7310103; doi:10.1186/s12906-020-02996-3)

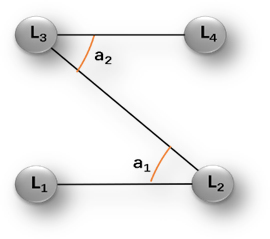

Supplement: Supplementary file 2 — Additional file 2: Figure S1. Diagram for the calculation of joint angles. [file 12906_2020_2996_MOESM2_ESM.jpg]

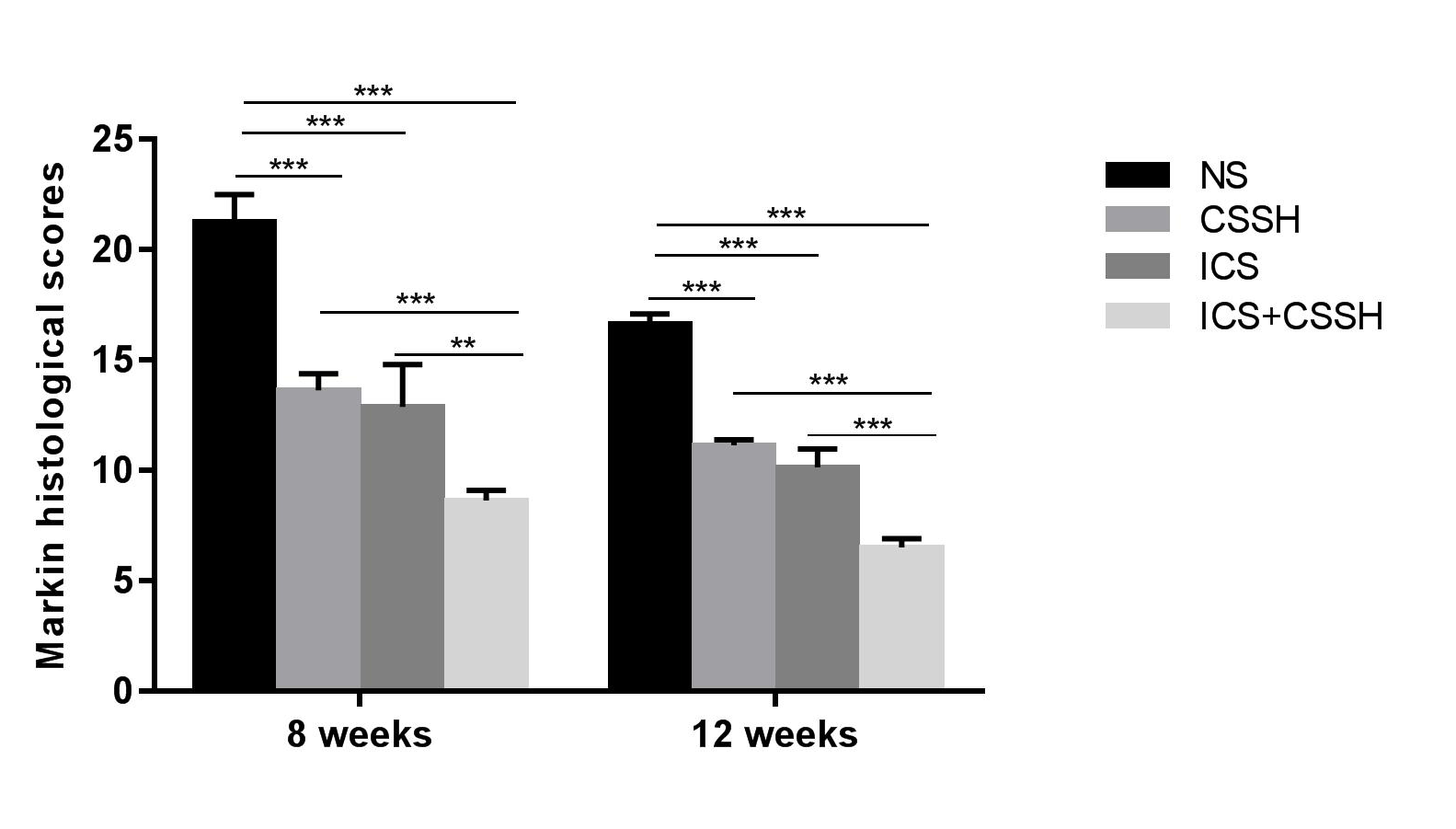

Supplement: Supplementary file 3 — Additional file 3: Figure S2. Markin scores for the evaluation of H&E staining in NS, CSSH, ICS and ICS-CSSH groups. *P < 0.05, **P < 0.01 vesus indacated groups. [file 12906_2020_2996_MOESM3_ESM.tif]
